# Comparative phylogenomic analyses of teleost fish Hox gene clusters: lessons from the cichlid fish Astatotilapia burtoni: comment

**DOI:** 10.1186/1471-2164-9-35

**Published:** 2008-01-24

**Authors:** Morgane Thomas-Chollier, Valérie Ledent

**Affiliations:** 1 Belgian EMBnet Node, Université Libre de Bruxelles – CP 257, Bd du Triomphe, B-1050 Brussels, Belgium; 2 Genomes and Networks Bioinformatics, Université Libre de Bruxelles – CP 263, Bd du Triomphe, B-1050 Brussels, Belgium; 3 Laboratory for Cell Genetics, Vrije Universiteit Brussel, Pleinlaan 2, B-1050 Brussels, Belgium

## Abstract

A reanalysis of the sequences reported by Hoegg *et al *has highlighted the presence of a putative HoxC1a gene in *Astatotilapia burtoni*. We discuss the evolutionary history of the HoxC1a gene in the teleost fish lineages and suggest that HoxC1a gene was lost twice independently in the Neoteleosts. This comment points out that combining several gene-finding methods and a Hox-dedicated program can improve the identification of Hox genes.

## Background

The identification of individual Hox genes is an essential basis for their study in evolutionary research fields. It is even more important in teleost fish where the unravelling of zebrafish and pufferfish Hox clusters have contributed to the establishment of the fish-specific genome duplication hypothesis. In a recent study, Hoegg *et al *[1] present the Hox gene content of the cichlid fish *Astatotilapia burtoni*, characterized from the complete sequence of the seven Hox clusters. Availability of these sequences is extremely valuable to help the community better understand the evolutionary plasticity of Hox genes and their regulatory elements in teleosts. A total of 46 Hox coding sequences have been identified, using common gene detection methods relying on sequence similarity. Identification of Hox multigenic family members is hampered by the high conservation of the homeodomain. We believe that a set of complementary approaches is thus required to correctly annotate all members of the Hox family.

Here, we complement the analysis of the Hox gene content of *Astatotilapia burtoni *reported in [1], using a combination of sequence similarity methods, *de-novo *gene predictions and a program we have developed that specifically classifies Hox proteins in their homology groups [2]. In addition, we collect a comprehensive set of HoxC1a sequences that allows us to re-investigate the presence of HoxC1a pseudogenes in various teleost species. In light of our findings, we discuss a revised version of HoxC1a gene loss events in teleost lineages.

## Results and Discussion

### HoxC1a gene detection

*Astatotilapia burtoni *Hox cluster genomic sequences were collected from GenBank and submitted to the *de-novo *gene prediction program GENSCAN [3]. A total of 102 putative coding sequences were localized on the genomic sequences. We applied HoxPred [2] on each putative peptide, and 37 sequences were predicted as Hox proteins. We have also applied HoxPred on the protein sequences detected by Hoegg *et al *and the resulting classification in homology groups is concordant with their result in both cases. We observed that GENSCAN predictions sometimes encompass two or three genes in a single predicted peptide. This sole method thus detects less Hox genes than reported in [1].

With this method, we have located a paralogous group (PG) 1 prediction on the HoxCa cluster. A detailed analysis of the predicted gene shows that GENSCAN, and other *de-novo *gene prediction programs, fail to correctly predict the C-terminal portion of its homeodomain. Alignment of zebrafish HoxC1a peptide to the HoxCa cluster with GeneWise (global mode) [4] supports the prediction of the first exon and completes the homeodomain sequence. The resulting putative peptide (Additional file 1) comprises two exons and one intron, its length is 295 residues and its genomic position downstream of HoxC3a (Additional file 2) strongly suggest a potential HoxC1a. Expression data would be needed to support this prediction.

HoxPred has previously been applied on the teleost fish *Gasterosteus aculeatus *proteome to characterise its Hox gene content [2]. We have shown that this fish comprises a putative HoxC1a gene partially supported by EST evidence. We performed pairwise alignments between full-length HoxC1a protein sequences detected in teleosts. The Neosteleost *A. burtoni *and *G. aculeatus *HoxC1a are very similar with 66% identity. On the contrary, *A. burtoni *HoxC1a only shares 30% identity with the Ostariophysii zebrafish HoxC1a, in the homeodomain for the most part.

### Phylogenetic analyses in paralogous group 1

Phylogenetic reconstructions of PG1 homeodomains from teleost species were conducted as in [2], with the addition of *A. burtoni *and *Fundulus heteroclitus *sequences. The HoxC1a sequence recently reported in the Ostariophysii *Megalobrama amblycephala *[5] was not included as the PCR fragment does not comprise the homeodomain.

**Figure 1:**
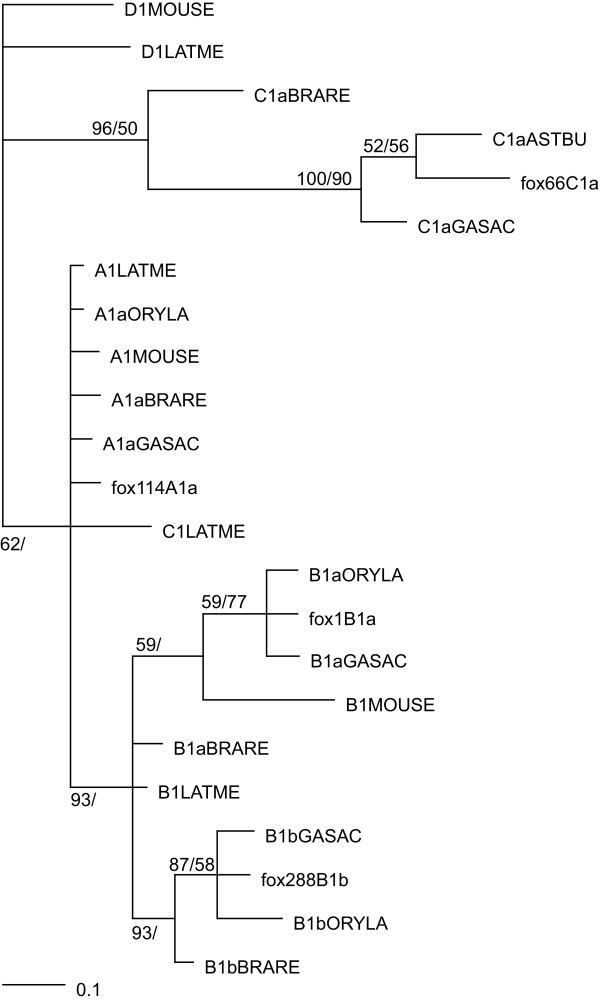
**Phylogenetic tree of Paralogous group 1 in selected vertebrates**. Phylogenetic tree reconstructions were conducted with homeodomain sequences as in [2]. The represented tree is obtained by bayesian inference (BI) using MrBayes [11] [12]. Rooting is arbitrary. The first numbers above the internal branches are posterior probabilities obtained by BI. The second numbers correspond to bootstrap values produced by the program PHYML of maximum-likelihood (ML) tree reconstruction [13]. Only statistical support values > 50 for at least one of the methods used (ML or BI) are shown. Marginal probabilities at each internal branches were taken as a measure of statistical support. All the alignements and the trees are available upon request. Abbreviations: LATME: Latimeria menadoensis, BRARE: Danio rerio, ASTBU: Astatotilapia burtoni, GASAC: Gasterosteus aculeatus, fox: Fundulus heteroclitus, ORYLA: Oryzia latipes.

Phylogenetic analyses confirm that *Astatotilapia burtoni *comprises a putative HoxC1a gene (Figure 1). In the frame of novel HoxC1a sequences, these phylogenetic tree reconstructions refine the analysis of *F. heteroclitus *PG1 PCR fragments [6] and provide additional evidence to confirm the classification previously proposed in [7].

### Fishing HoxC1a pseudogenes out of teleost genomes

The Neoteleosts HoxC1a genes we have identified provide a more comprehensive set of sequences that can be used to investigate HoxC1a pseudogenes in teleost sequences. We have analysed the region downstream HoxC3a of the medaka *Oryzia latipes *in search of a putative HoxC1a gene. The EnsEMBL [8] GENSCAN prediction that spans this region did not return any potential Hox gene with HoxPred. GeneWise alignments of *O. latipes *genomic sequence with *G. aculeatus *and *A. burtoni *HoxC1a proteins nevertheless highlight a genomic region in *O. latipes*, corresponding to both N- and C-terminal portions of the proteins (see Additional file 2 for genomic positions). As the homeodomain is highly degenerate and contains a frameshift mutation, we argue in favor of a HoxC1a pseudogene in medaka.

We performed similar analyses on the genomic sequences of pufferfishes (*Takifugu rubripes *and *Tetraodon nigroviridis*) and observed imprints of HoxC1a pseudogenes in both cases. For *T. rubripes*, this finding is in agreement with the HoxC1a pseudogene described in [9]. For *T. nigroviridis*, no HoxC1a pseudogene has been reported yet, and a previous attempt to identify a functional HoxC1a gene was unsuccessful [7].

We have constructed mVista plots [10] as performed in [1] to highlight conserved non-coding sequences downstream of HoxC3a with a comparative genomic approach (Additional file 3). As previously noted by Hoegg *et al*, we observe a high similarity between *G. aculeatus *and *A. burtoni*. This plot also shows a high similarity between the Neoteleost pseudogenes and putative genes, whereas zebrafish HoxC1a sequence is clearly less similar.

### HoxC1a gene loss in the teleost Hox clusters

**Figure 2:**
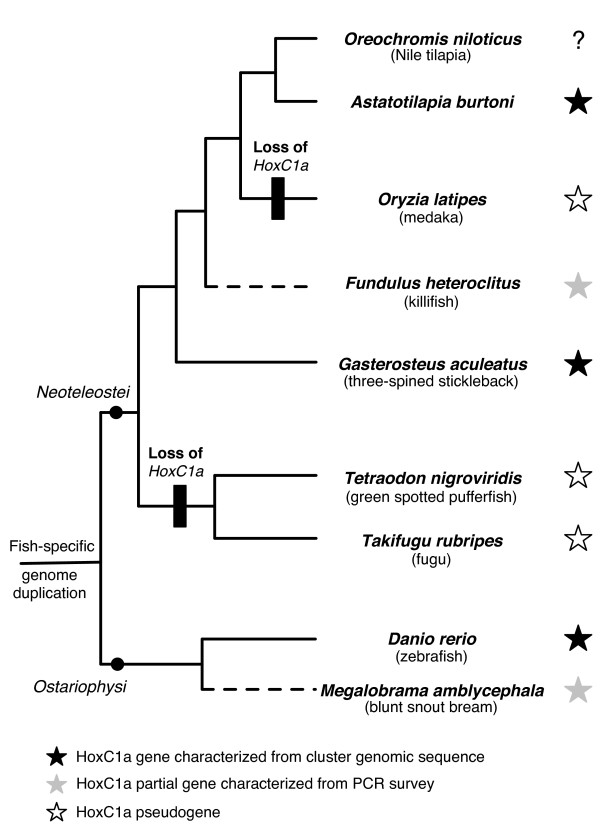
**Overview of HoxC1a content in teleost species and gene loss events mapped on a phylogeny**. HoxC1a genes are depicted with stars. Dashed lines indicate that corresponding species were not reported in [1] and their position in the phylogeny is hypothetic.

Based on the sole presence of HoxC1a gene in zebrafish, Hoegg *et al *suggest that HoxC1a has been lost once in the lineage leading to Neoteleosts (as illustrated in figure 3 in [1]). Presence of this gene in both *G. aculeatus *and *A. burtoni *rejects this hypothesis. Figure 2 is a comprehensive overview of the current HoxC1a set of orthologs in teleosts, according to our results based on publicly available data. We have mapped HoxC1a gene loss events on the phylogeny reported in [1]. It indicates that HoxC1a has been lost independently among Neoteleosts, in both lineages leading to *O. latipes *and to the pufferfishes. Whether an additional HoxC1a gene loss has occurred in the lineage leading to the cichlid fish *Oreochromis niloticus *remains to be investigated.

## Conclusion

*A. burtoni *was reported to contain 46 Hox genes. We have complemented the Hox gene content of this fish with a putative HoxC1a gene. Combined with the detection of HoxC1a orthologs in *G. aculeatus *and *F. heteroclitus*, we introduce here a more comprehensive set of HoxC1a genes in teleosts. These Neoteleost genes facilitate the investigation of pseudogenes in *O. latipes *and pufferfishes in comparison with the more distant zebrafish ortholog. We report two novel HoxC1a pseudogenes, in *O. latipes *and *T. nigroviridis *respectively. In addition, this case-study illustrates the annotation challenge posed by the Hox multigenic family. We have shown that Hox identification can be improved by combining several gene-finding methods and a Hox-dedicated program.

This comment has hopefully given new insights into the gene loss events presented by Hoegg *et al*, as regards to HoxC1a. Our results modify their conclusions and rule out the hypothesis of a unique HoxC1a gene loss event in the lineage leading to Neoteleosts. We propose that HoxC1a was independently lost in the lineage leading to *O. latipes *and in the lineage leading to pufferfishes. Our findings do not affect other aspects of the Hoegg *et al *study, especially the fact that each teleost species studied so far contains a different Hox gene set. Rather, we believe that this contribution reinforces their conclusions about non-essential Hox genes that can be easily and repeatedly lost like HoxB7a or HoxC1a.

## Authors' contributions

MT-C conceived the study, performed the sequence analyses and drafted the manuscript. VL performed the phylogenetic analyses and participated in the editing of the manuscript. All the authors read and approved the final manuscript.

## Supplementary Material

Additional file 1 Putative HoxC1a protein sequences. The data provided represent the HoxC1a protein sequences predicted in selected teleosts, in Fasta format. Click here for file

Additional file 2 Genomic coordinates of putative HoxC1a genes and pseudogenes. The data provided represent the genomic positions of the predicted HoxC1a genes and pseudogenes in selected teleosts. Click here for file

Additional file 3 mVista plot downstream of the HoxC3a gene. Figure showing the evolutionary conserved regions downstream of the HoxC3a gene, in selected teleosts. Click here for file
