# Supplementary figures and images for: Comparative phylogenomic analyses of teleost fish Hox gene clusters: lessons from the cichlid fish Astatotilapia burtoni: comment
Source: BMC Genomics. 2008 Jan 24;9:35. doi: 10.1186/1471-2164-9-35 (PMC2246111; doi:10.1186/1471-2164-9-35)

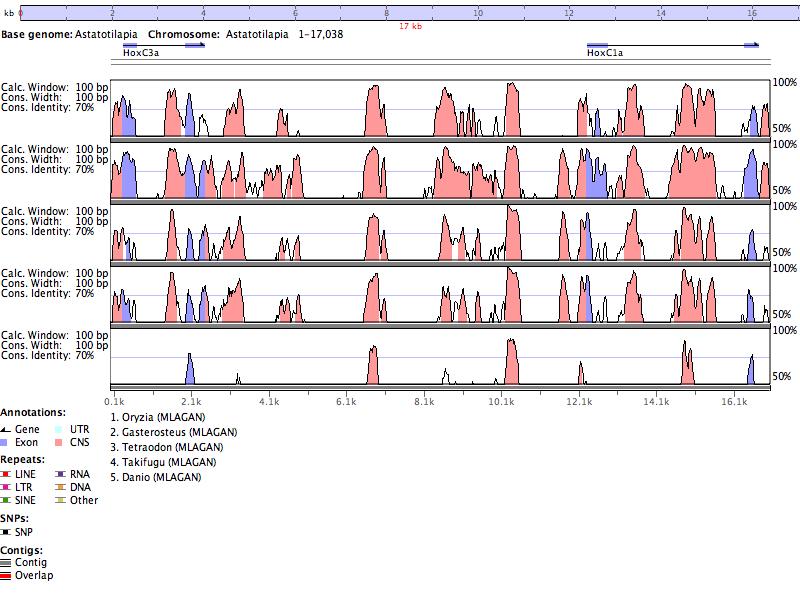

Supplement: Additional file 3 — mVista plot downstream of the HoxC3a gene. Figure showing the evolutionary conserved regions downstream of the HoxC3a gene, in selected teleosts. [file 1471-2164-9-35-S3.jpeg]
